# From dysbiosis to decolonization: The emerging role of fecal microbiota transplantation in antimicrobial resistance

**DOI:** 10.1016/j.imj.2026.100253

**Published:** 2026-04-21

**Authors:** Timotius Ivan Hariyanto

**Affiliations:** a Department of Microbiology, Faculty of Medicine, Universitas Airlangga, Surabaya, 60132, Indonesia; b Department of Microbiology, Dr. Soetomo General Hospital, Surabaya, 60132, Indonesia

Antimicrobial resistance (AMR) is steadily eroding the effectiveness of the very drugs that have underpinned modern medicine for decades.^1^ Recent global estimates suggest that millions of deaths each year are associated with drug-resistant bacterial infections, with particularly steep increases for pathogens such as methicillin-resistant *Staphylococcus aureus* and multidrug-resistant Gram-negative organisms.^1^ In many regions, roughly one in six bacterial infections now involves a resistant pathogen, and in some clinical syndromes, resistant strains account for up to one in three cases.^1^ These trends translate into longer hospital stays, higher healthcare expenditures, and constrained options for procedures that rely on effective prophylaxis, including chemotherapy, transplantation, and major surgery.^1^^,^^2^

Despite intensified stewardship and infection prevention measures, managing AMR at the bedside often feels like trying to contain a fire with diminishing water supplies.^2^ Clinicians are forced to choose between toxic last-line agents, prolonged combination regimens, and off-label use of newer antimicrobials, all against a background of fragile evidence and escalating costs.^2^^,^^3^ Colonization with multidrug-resistant organisms (MDROs) in the gut has emerged as a particular challenge, especially in immunocompromised patients, where intestinal carriage frequently precedes invasive bloodstream infection and can drive repeated treatment courses.^3^ Traditional decolonization strategies that rely on non-absorbable antibiotics risk further perturbing the microbiome and may paradoxically amplify selection pressure for resistance.^3^

Against this backdrop, fecal microbiota transplantation (FMT) has attracted attention as a fundamentally different strategy: Instead of escalating antibiotic intensity, it attempts to restore a resilient microbial ecosystem capable of suppressing MDROs.^4^^,^^5^ FMT, established as an effective therapy for recurrent *Clostridioides difficile* infection, involves administering processed stool from carefully screened healthy donors to recipients via colonoscopy, nasoenteric routes, enema, or increasingly, oral capsules containing frozen or lyophilized material.^4^^,^^5^ The conceptual shift is notable—away from targeting individual resistant species and toward repairing the community-level structure of the gut microbiome that ordinarily constrains pathogen overgrowth.^4^^,^^5^ Yet, even as enthusiasm grows, it is important to recognize that current evidence remains preliminary, with major questions about scalability, durability of benefit, and the balance between decolonization and safety still unresolved.

The PREMIX randomized controlled trial provides some of the clearest evidence to date that FMT can accelerate MDRO decolonization in a high-risk population.^6^ In this study of renal transplant recipients with documented MDRO infections and persistent gut colonization (primarily extended-spectrum β-lactamase [ESBL]–producing *Enterobacterales*), participants were randomized to bowel preparation alone or bowel preparation followed by FMT delivered as a retention enema.^6^ By day 36 of the first cycle, all participants in the observation arm (5/5) remained MDRO-positive on stool culture, whereas only one-third of those who received FMT (2/6) still carried MDROs.^6^ When the full protocol was completed, 8 of 10 evaluable participants (80%) who received at least one FMT—and 8 of 9 (89%) who received all planned FMTs—were MDRO-negative at their final visit.^6^ Time to negative stool culture was significantly shorter with FMT than with observation (median 35 vs. 88 days), and in a post hoc comparison against contemporaneous renal transplant controls, FMT-treated patients experienced a longer time to recurrent MDRO infection over 180 days.^6^

Complementary mechanistic analyses from PREMIX demonstrated that these clinical effects coincided with meaningful shifts in microbiome structure and function.^6^ FMT increased alpha diversity in treated participants, while the abundance of antimicrobial resistance genes decreased significantly compared with baseline.^6^ Metagenomic and metabolomic profiling showed that responders moved along two distinct ecological trajectories, with some patients exhibiting profound dysbiosis at baseline that “normalized” after FMT, while others with milder disruption showed more modest but still favorable shifts.^6^ Importantly, the trial revealed a previously underappreciated mechanism of MDRO control: Conspecific strain replacement.^6^

In PREMIX, high-resolution sequencing of isolates and stool metagenomes uncovered cases in which highly resistant ESBL-producing *Enterobacterales* strains were replaced by more susceptible strains of the same species after FMT.^6^ In three participants who ultimately cleared MDRO colonization, non-ESBL conspecific strains with broader antibiotic susceptibility profiles emerged and persisted while the original resistant strains disappeared.^6^ Strain-level analyses indicated that these susceptible strains were not acquired from the donor product but were often present at low abundance in the recipient before FMT, expanding only once the ecological conditions in the gut shifted.^6^ This suggests that FMT may not simply “wipe out” MDROs; rather, it may support expansion of pre-existing, more susceptible competitors that displace dominant resistant clones through niche competition.^6^

However, the strength of inferences that can be drawn from PREMIX is limited by several design features that are often under-emphasized in narrative discussions.^6^ The total sample size was very small, with only 11 randomized participants and 10 ultimately evaluable, which inflates the risk of chance findings and makes effect-size estimates inherently unstable.^6^ PREMIX was also conducted at a single center in a narrowly defined population of renal transplant recipients with specific MDRO profiles, raising questions about external validity when extrapolating to other immunocompromised hosts, different MDRO spectra, or general medical inpatients.^6^ Follow-up was relatively short, and although decolonization at the end of study and reduced recurrent infections at 180 days are encouraging, long-term data on recolonization, resistance evolution, and hard outcomes (such as mortality or cumulative antibiotic exposure over years) remain lacking.^6^ Taken together, PREMIX should be viewed as an important proof-of-concept trial rather than definitive evidence that FMT-based decolonization is ready for broad implementation.

The broader Sentinel REACT nonrandomized trial, conducted in a long-term acute care hospital, adds a complementary perspective by focusing on feasibility, safety, and infection outcomes in a severely ill, highly colonized cohort.^7^ In this pilot study, 10 patients with intestinal MDRO colonization received a single donor-derived FMT via gastrostomy tube or enema and were compared with 32 contemporaneous MDRO-colonized controls.^7^ FMT was acceptable to patients and caregivers, with no serious adverse events attributed to the product and only mild, self-limited gastrointestinal symptoms in the days following treatment.^7^ Although all FMT recipients remained culture-positive for at least one MDRO at follow-up (reflecting the difficulty of achieving complete eradication in this setting), exploratory analyses suggested potential clinical benefits: No FMT recipients developed positive blood cultures in the six months after treatment, compared with 19% of controls, and FMT recipients had numerically fewer days of antibiotic therapy per 1000 patient-days, although these differences did not reach statistical significance in this small pilot.^7^

The Sentinel REACT data also illustrated that even when MDRO cultures remain positive, FMT can increase microbial diversity and reduce intestinal pathogen dominance, which has been linked in other studies to lower risks of bloodstream and urinary infections.^7^ Species-level alpha diversity rose in FMT recipients over 28 days, while it declined in untreated controls; simultaneously, the proportion of patients with microbiome “domination” by a single pathogen genus decreased among FMT recipients but increased among controls.^7^ These patterns support a model in which partial ecological restoration after FMT reduces pathogen burden to levels less likely to lead to invasive infection, even when colonization is not completely eradicated.^7^

Yet, like PREMIX, Sentinel REACT illustrates both the promise and the current fragility of the evidence base.^7^ The study’s nonrandomized design introduces the possibility of selection bias and uncontrolled confounding, particularly in a complex long‑term acute care population where clinical trajectories and infection risks are shaped by multiple co‑interventions.^7^ The intervention was again evaluated in a single institution, which may have specific microbiological epidemiology, infection‑control practices, and patient characteristics that are not representative of other settings.^7^ Sample size was modest, especially in the FMT arm, and follow‑up was limited to six months, leaving unanswered questions about sustained effects on colonization, resistance gene dynamics, and downstream antimicrobial consumption.^7^ These features mean that the apparent reduction in bloodstream infections and antibiotic days should be interpreted as hypothesis‑generating rather than definitive evidence of clinical benefit.

Beyond these trials, emerging observational data further explore FMT for MDRO decolonization in the gut. A recent cohort study reported that FMT delivered as frozen oral capsules in patients colonized with carbapenem‑resistant *Enterobacterales* or vancomycin‑resistant *Enterococci* was associated with higher rates of decolonization compared with historical controls receiving standard care, particularly when multiple FMT courses were administered.^8^ Another investigation in hematology‑oncology and solid organ transplant recipients suggested that FMT could reduce carriage of extended‑spectrum β‑lactamase–producing organisms and carbapenem‑resistant strains, with some patients achieving sustained clearance beyond three to six months.^9^ These newer reports, although heterogeneous in design and often limited by small sample sizes and lack of randomization, reinforce the signal that FMT can modify MDRO colonization trajectories in real‑world high‑risk populations.^8^^,^^9^ At the same time, they underscore persistent uncertainties about optimal dosing, route, and the proportion of patients who derive durable benefit versus transient or no effect.

Additional microbiome-focused work has helped clarify why FMT may be uniquely suited to address MDRO colonization.^10^ A translational trial examining FMT in MDRO-colonized patients showed that FMT not only reduced the abundance of resistance genes but also reshaped metabolic outputs, including short-chain fatty acids and bile acid profiles, in ways that likely support colonization resistance.^10^ In PREMIX, participants with the most disrupted baseline microbiomes experienced the largest gains in metabolites such as butyrate and isovalerate after FMT, alongside decreases in glycocholate, a bile acid associated with enhanced *Escherichia coli* pathogenicity.^6^ Donor taxa that frequently engrafted in responders included *Akkermansia muciniphila, Faecalibacterium prausnitzii, Barnesiella intestinihominis*, and other anaerobes known or suspected to contribute to mucosal health, immune modulation, and competitive exclusion of pathogens.^6^

Taken together, these findings suggest several plausible mechanisms by which FMT can promote MDRO decolonization or risk reduction. First, restoration of a diverse anaerobic community reinforces competitive exclusion, with commensals outcompeting MDROs for nutrients and mucosal niches.^6^^,^^7^^,^^11^^,^^12^ Second, enhanced production of short-chain fatty acids and other metabolites improves epithelial barrier integrity and shapes local immune responses, which may limit translocation of Gram-negative organisms into the bloodstream.^6^^,^^7^^,^^11^ Third, strain-level competition within species—where susceptible strains displace resistant ones—provides an elegant route to reducing the clinical impact of MDRO colonization without necessarily eradicating the species entirely.^6^^,^^7^^,^^11^ Finally, reductions in the overall “resistome” of the gut microbiota may lessen the pool of resistance genes available for horizontal transfer.^6^^,^^7^^,^^12^

However, it is crucial to emphasize that most mechanistic insights to date are correlational rather than demonstrably causal. In both PREMIX and Sentinel REACT, microbiome changes and clinical events are temporally linked, but direct causal pathways—showing that specific microbial taxa, metabolic signatures, or strain-replacement patterns independently drive infection risk reduction—have not yet been firmly established.^6^^,^^7^ Key unresolved questions include how to best match donors and recipients, which baseline features predict engraftment and successful decolonization, and whether certain “keystone” species or functional modules are necessary for durable benefit. The field also lacks robust biomarkers that can guide real-time assessment of whether FMT has meaningfully shifted a recipient’s microbiome toward a less permissive state for MDROs. Answering these questions will be essential if FMT is to transition from a largely empirical intervention to a more precisely targeted therapy.

Yet, the question remains: can FMT truly be considered a new hope for lowering AMR at scale, or is it better viewed as a targeted tool for selected high-risk situations? Current evidence supports cautious optimism rather than unqualified enthusiasm.^5^, ^6^, ^7^, ^8^, ^9^, ^10^ On the one hand, the observation that FMT can reduce MDRO colonization in a substantial proportion of patients, and that decolonization appears to correlate with fewer MDRO bloodstream infections, is encouraging.^6^^,^^7^ The ability to leverage microbiome restoration instead of additional antibiotics aligns with global calls to reduce antimicrobial use and preserve existing agents.^6^^,^^7^ On the other hand, heterogeneity in decolonization rates, small sample sizes, and variations in methodology limit the generalizability of available data.^5^, ^6^, ^7^, ^8^, ^9^, ^10^

Furthermore, FMT is not without risk. Although serious adverse events appear infrequent, there have been safety warnings related to potential transmission of unrecognized pathogens or resistance genes through donor stool, emphasizing the importance of stringent donor screening and standardized manufacturing processes.^13^^,^^14^ Regulatory frameworks are still evolving, and the logistics of donor recruitment, material processing, and delivery can be challenging outside specialized centers.^13^^,^^14^ From a health systems perspective, FMT is unlikely to replace conventional infection prevention and antimicrobial stewardship strategies; instead, it should be integrated as an adjunctive approach for select patients in whom MDRO colonization is clearly linked to recurrent severe infections and limited treatment options.^6^, ^7^, ^8^, ^9^, ^10^ In practical terms, this means careful patient selection (for example, heavily colonized transplant or hematology‑oncology patients with repeated MDRO infections), thoughtful timing (such as after stabilization from an acute infection and de‑escalation of broad‑spectrum antibiotics), and a realistic appraisal of feasibility in non‑specialized settings where access to donor material, endoscopy, and advanced microbiome testing may be constrained.

Looking ahead, several directions for research and clinical practice seem particularly urgent, but not all are equally translatable in the near term. One high‑priority area is the development and rigorous evaluation of standardized oral FMT capsule formulations that can be deployed safely and reproducibly across centers, including those without ready access to endoscopy or specialized microbiome laboratories. Such products—ideally produced under good manufacturing practice conditions with harmonized donor screening and batch characterization—could substantially lower the logistical barriers to integrating FMT into routine care while also facilitating large multicenter trials.^13^^,^^14^ A second priority is the transition from heterogeneous stool preparations to more defined microbial communities or consortia that retain the ecological functions necessary for MDRO suppression but reduce the risk of transmitting unwanted organisms or resistance elements.^13^^,^^14^ In parallel, prospective studies should systematically monitor not only individual MDROs but also broader “resistance groups” and resistome dynamics over time, in order to understand how FMT and related interventions influence the trajectory of AMR at the patient and ward level.

Importantly, any expansion of FMT for MDRO decolonization will need to be embedded within existing infection‑prevention and antimicrobial‑stewardship frameworks rather than operating in isolation. FMT should be considered when standard measures—such as contact precautions, environmental cleaning, catheter management, and optimized antibiotic prescribing—have been fully implemented yet patients remain at high risk of recurrent MDRO infections. Stewardship teams can help identify appropriate candidates, align FMT timing with efforts to de‑escalate or discontinue broad‑spectrum antibiotics, and ensure that FMT does not inadvertently become a justification for more liberal antimicrobial use. Infection‑prevention teams, in turn, will need to work with clinicians to determine how decolonization data—especially partial or transient reductions in carriage—should influence isolation policies and cohorting strategies. Implementing FMT programs outside specialized centers will also require attention to pragmatic issues, including training, supply chains for oral capsules or frozen preparations, reimbursement, and clear communication with patients about realistic expectations and residual uncertainties.

In summary, FMT represents a compelling example of how microbiome‑based therapies might be harnessed to confront AMR by reshaping ecological landscapes rather than escalating pharmacologic pressure on pathogens. Early data suggest that FMT can meaningfully reduce MDRO colonization and related infections in selected high‑risk patients, but outcomes are variable and the evidence base remains emergent, with major gaps in sample size, diversity of settings, mechanistic understanding, and long‑term follow‑up.^5^, ^6^, ^7^, ^8^, ^9^, ^10^ At present, FMT should be considered a promising, yet still investigational, adjunct in the broader fight against AMR rather than a stand‑alone solution. With rigorous multicenter trials, standardized and safer microbiome products, better mechanistic biomarkers, and deliberate integration into comprehensive stewardship and infection‑prevention programs, FMT and related microbiome interventions may evolve from experimental rescue therapies into structured tools that contribute—alongside conventional measures—to bending the curve of antimicrobial resistance.

## CRediT authorship contribution statement

**Timotius Ivan Hariyanto:** Conceptualization, Investigation, Supervision, Visualization, Writing – original draft, Writing – review & editing.

## Informed consent

Not applicable.

## Organ donation

Not applicable.

## Ethical statement

Not applicable.

## Data availability statement

No new data were generated or analyzed. Data sharing is not applicable to this article.

## Animal treatment

Not applicable.

## Generative AI

Artificial intelligence tools were used solely for language refinement and paraphrasing purposes. No AI tools were used to generate original scientific content, interpretations, or conclusions.

## Funding

None.

## Declaration of competing interest

The authors declare that they have no known competing financial interests or personal relationships that could have appeared to influence the work reported in this paper.

## References

[1] Naghavi M., Vollset S.E., Ikuta K.S. (2024). Global burden of bacterial antimicrobial resistance 1990–2021: a systematic analysis with forecasts to 2050. Lancet.

[2] Patra M., Gupta A., Kumar D. (2025). Antimicrobial resistance: a rising global threat to public health. Infect Drug Resist.

[3] Ling Z.X., Ding W.W., Liu X. (2025). Gut microbiota dysbiosis and systemic immune dysfunction in critical ill patients with multidrug-resistant bacterial colonization and infection. J Transl Med.

[4] Alagna L., Palomba E., Mangioni D. (2020). Multidrug-resistant gram-negative bacteria decolonization in immunocompromised patients: a focus on fecal microbiota transplantation. Int J Mol Sci.

[5] Hyun J., Lee S.K., Cheon J.H. (2022). Faecal microbiota transplantation reduces amounts of antibiotic resistance genes in patients with multidrug-resistant organisms. Antimicrob Resist Infect Control.

[6] Woodworth M.H., Conrad R.E., Haldopoulos M. (2023). Fecal microbiota transplantation promotes reduction of antimicrobial resistance by strain replacement. Sci Transl Med.

[7] Woodworth M.H., Babiker A., Prakash-Asrani R. (2025). Microbiota transplantation among patients receiving long-term care: the sentinel REACT nonrandomized clinical trial. JAMA Netw Open.

[8] Shin J., Lee J.H., Park S.H. (2022). Efficacy and safety of fecal microbiota transplantation for clearance of multidrug-resistant organisms under multiple comorbidities: a prospective comparative trial. Biomedicines.

[9] Kaźmierczak-Siedlecka K., Skonieczna-Żydecka K., Biliński J. (2021). Gut microbiome modulation and faecal microbiota transplantation following allogenic hematopoietic stem cell transplantation. Cancers.

[10] Innes A.J., Mullish B.H., Ghani R. (2021). Fecal microbiota transplant mitigates adverse outcomes seen in patients colonized with multidrug-resistant organisms undergoing allogeneic hematopoietic cell transplantation. Front Cell Infect Microbiol.

[11] Tang B.F., Cao Y., Li J.S. (2025). Whole microbiota transplantation restores gut homeostasis throughout the gastrointestinal tract. Imeta.

[12] Sahu K., Satapathy T., Sahu P. (2026). Nanocarrier-induced inflammation: mechanisms, immunometabolic impacts and strategies for safer design. TransMed.

[13] Mehta N., Wang T., Friedman-Moraco R.J. (2022). Fecal microbiota transplantation donor screening updates and research gaps for solid organ transplant recipients. J Clin Microbiol.

[14] Goldsmith J., Tomkovich S., Auniņš J.G. (2024). End-to-end donor screening and manufacturing controls: complementary quality-based strategies to minimize patient risk for donor-derived microbiome therapeutics. Gut Microbes.

